# Constraint and Adaptation in newt Toll-Like Receptor Genes

**DOI:** 10.1093/gbe/evu266

**Published:** 2014-12-04

**Authors:** Wiesław Babik, Katarzyna Dudek, Anna Fijarczyk, Maciej Pabijan, Michał Stuglik, Rafał Szkotak, Piotr Zieliński

**Affiliations:** Institute of Environmental Sciences, Jagiellonian University, Kraków, Poland

**Keywords:** amphibia, Approximate Bayesian Computation, *Lissotriton*, positive selection, Toll-like receptors

## Abstract

Acute die-offs of amphibian populations worldwide have been linked to the emergence of viral and fungal diseases. Inter and intraspecific immunogenetic differences may influence the outcome of infection. Toll-like receptors (TLRs) are an essential component of innate immunity and also prime acquired defenses. We report the first comprehensive assessment of TLR gene variation for urodele amphibians. The *Lissotriton* newt TLR repertoire includes representatives of 13 families and is compositionally most similar to that of the anuran *Xenopus*. Both ancient and recent gene duplications have occurred in urodeles, bringing the total number of TLR genes to at least 21. Purifying selection has predominated the evolution of newt TLRs in both long (∼70 Ma) and medium (∼18 Ma) timescales. However, we find evidence for both purifying and positive selection acting on TLRs in two recently diverged (2–5 Ma) allopatric evolutionary lineages (*Lissotriton montandoni* and *L. vulgaris graecus*). Overall, both forms of selection have been stronger in *L. v. graecus*, while constraint on most TLR genes in *L. montandoni* appears relaxed. The differences in selection regimes are unlikely to be biased by demographic effects because these were controlled by means of a historical demographic model derived from an independent data set of 62 loci. We infer that TLR genes undergo distinct trajectories of adaptive evolution in closely related amphibian lineages, highlight the potential of TLRs to capture the signatures of different assemblages of pathogenic microorganisms, and suggest differences between lineages in the relative roles of innate and acquired immunity.

## Introduction

Plant and invertebrate genes involved in detection of pathogens tend to evolve rapidly. They are characterized by pervasive positive selection in *Drosophila* (Sackton et al. 2007) and by high gene turnover in multilocus families coupled with transient balancing selection in plants (Tiffin and Moeller 2006; Boller and He 2009). The need for high genetic diversity of innate sensors may have been reduced in vertebrates by transferring the role of the pathogen-specific response to the molecules of the adaptive immune system (Wlasiuk et al. 2009; Wlasiuk and Nachman 2010). However, as evidenced by the diversity of the vertebrate innate immune system (Akira et al. 2006; Hansen et al. 2011; Kawai and Akira 2011), rapid recognition of conserved molecular patterns associated with pathogens is still essential.

Toll-like receptors (TLRs) are pattern recognition receptors that sense conserved pathogen associated molecular patterns (PAMPs; Akira et al. 2006; Kawai and Akira 2010). TLRs are of paramount importance for the innate immune response and also provide a crucial link between innate and adaptive immune systems (Iwasaki and Medzhitov 2010; Kawai and Akira 2010). Vertebrates usually have a limited number of TLRs specialized in recognition of various PAMPs (Palti 2011; Keestra et al. 2013). These *TLR* genes form several families (Roach et al. 2005; Temperley et al. 2008) and representatives of most families appear indispensable for an effective immune response (Medzhitov 2001; Takeda et al. 2003).

Evolutionary rates in various *TLR* families have been reported as similar and slow (Roach et al. 2005; Temperley et al. 2008). Within the molecules, the extracellular part, consisting of multiple leucine-rich repeats (LRR), is involved in pathogen recognition and evolves faster than the intracellular Toll/interleukin-1 receptor (TIR) domain responsible for intracellular signaling (Mikami et al. 2012). However, the image of a static, functionally conserved and slowly evolving *TLR* may be oversimplified as suggested by interspecific differences in ligand recognition (Werling et al. 2009; Palti 2011) and by major differences in *TLR* repertoires among vertebrate groups (Roach et al. 2005; Temperley et al. 2008). Moreover, observations of multiple instances of recurrent and episodic positive selection occurring in various taxonomic groups on relatively short timescales (Wlasiuk et al. 2009; Wlasiuk and Nachman 2010; Alcaide and Edwards 2011; Tschirren et al. 2011) are inconsistent with slowly evolving and strongly conserved *TLR* genes. Associations between *TLR* polymorphism and susceptibility to infection have been demonstrated in rodent (Tschirren et al. 2013) and human (Netea et al. 2012) populations, again suggesting that episodes of positive selection in *TLR* genes may be prompted by changes in PAMP or in the composition of pathogen communities. Thus, *TLR* genes are substantially conserved, but episodic positive as well as transient balancing selection are not uncommon and variation segregating in populations may be of adaptive significance (Tschirren et al. 2012, 2013).

A detailed study of *TLR* polymorphism in human populations revealed variable levels of constraint, abundant polymorphism and recent positive selection at some loci (Ferrer-Admetlla et al. 2008; Barreiro et al. 2009; Mukherjee et al. 2009, 2014; Wlasiuk and Nachman 2010). The most constrained human TLRs are *03*, *07*, *08*, and *09* which act as sensors of pathogen nucleic acids (NAs). Barreiro et al. (2009) suggested that less constrained TLRs, in which missense or even nonsense mutations are tolerated, may be promising targets of transient positive/balancing selection because polymorphisms segregating in populations for longer periods of time should be available for selection if environmental conditions or pathogen pressure change. They also hypothesized that changes in pathogen pressure may render some innate immune receptor genes temporarily redundant. Such fluctuations in selection regimes could potentially explain the loss of certain *TLR* genes observed during vertebrate evolution and may also facilitate the evolution of altered ligand specificity.

Empirical evaluations of *TLR* polymorphism within and divergence between closely related species have been performed only in a handful of mammals and birds (Palermo et al. 2009; Downing et al. 2010; Grueber et al. 2012; Tschirren et al. 2012). The most comprehensive analyses include studies of humans (Barreiro et al. 2009) and apes (Wlasiuk and Nachman 2010; Quach et al. 2013), which demonstrated substantial interspecific differences in the strength of purifying selection acting on TLRs. Similar information is currently lacking for other vertebrate groups and for *TLR* genes absent in amniotes.

The only amphibians for which TLRs have been characterized so far are clawed frogs of the genus *Xenopus*. These anurans exhibit a *TLR* repertoire somewhat intermediate to that of fish and terrestrial vertebrates (Ishii et al. 2007). No data on the TLRs of urodele amphibians, a group which diverged from anurans about 300 Ma (Roelants et al. 2007), have been reported so far. This is unfortunate as amphibians are in a catastrophic decline partly attributed to emerging infectious diseases (Cheng et al. 2011; Fisher et al. 2012). Resistance to the most important of these diseases, chytridiomycosis, is contingent both on genetic differences among species (Woodhams et al. 2007) and variation within species (Savage and Zamudio 2011). TLRs, by providing a link between the innate and acquired branches of the immune system, may be particularly relevant to understanding the factors that affect susceptibility to chytridiomycosis (Richmond et al. 2009). Moreover, TLRs have been shown to mediate antifungal immunity in other vertebrates (Roeder et al. 2004; Luther and Ebel 2006). Given the acute need for comprehensive information on amphibian immunogenetics, we characterize the *TLR* repertoire and variation in urodeles with a main focus on newts of the genus *Lissotriton*. We use transcriptome sequences combined with high-throughput amplicon sequencing to identify, characterize, and assess polymorphism in newt *TLR* genes. We then identify selection pressures acting on the urodele *TLR* repertoire at long-term, medium-term, and recent evolutionary time scales. Specifically, we address the following questions: 1) what is the repertoire of *TLR* genes in urodeles and how does it compare to that of other vertebrate groups? 2) what is the extent of selective constraint of individual *TLR* genes in urodeles? Are there differences in constraint between amphibians and mammals, especially in NA-sensing and nonNA-sensing genes? 3) is there evidence of recent positive selection or polymorphisms of adaptive significance segregating in *TLR* genes in distinct evolutionary lineages of *Lissotriton* newts? 4) do overall patterns of selection on TLRs differ between lineages when controlling for historical demography?

## Materials and Methods

### Identification of the *TLR* Repertoire in the newt Transcriptome

Transcripts of *TLR* genes were identified in transcriptome assembled from liver and spleen mRNA of 14 newts. Liver transcriptomes of six *Lissotriton montandoni* and six *L. vulgaris* and spleen transcriptomes of two *L. vulgaris* were sequenced. Sequencing libraries were prepared using the TruSeq RNA kit starting from total RNA and sequenced with HiSeq2000 (Illumina). In total 471 million pairs (on average 33.6 ± [SD] 9.6 million per sample) of 100 bp reads were used for assembly with Trinity (Grabherr et al. 2011). A database of TLR protein sequences compiled from a diverse set of vertebrate species was used to identify *TLR* transcripts (using BLASTx algorithm) in the assembled transcriptome. The two *Lissotriton* species are closely related; due to ongoing hybridization and incomplete lineage sorting, they share a large number of polymorphisms, and in most gene trees the species are not reciprocally monophyletic (Zieliński et al. 2013; Pabijan M, Zieliński P, Babik W, unpublished data). Therefore we did not attempt to reconstruct *TLR* transcripts for each species; instead we used the assembled contig as a representative *L. montandoni/vulgaris TLR*-coding sequence for comparisons with other vertebrates. We did not analyze noncoding parts of the transcripts but took advantage of this information to design primers for an assessment of *TLR* polymorphism.

Transcripts of a number of *TLR* genes were identified in the newt transcriptome. Some of them were highly divergent from TLRs of other vertebrates. A phylogeny of vertebrate TLR proteins including those inferred from newt transcripts was used to assign newt genes to *TLR* families. This phylogeny included all major vertebrate *TLR* families, with several representatives from each to facilitate alignment. We searched the transcriptome derived from multiple tissues of the red-spotted newt (*Notophthalmus viridescens*) (Abdullayev et al. 2013) for orthologs of *Lissotriton TLR* genes and included them in the phylogenetic analysis if complete open reading frames (ORFs) could be identified. Alignment was done with MAFFT (Katoh et al. 2002) using the L-INS-i strategy and the BLOSUM45 substitution matrix. The best fitting model of amino acid substitution, the WGA model including rate variation among sites and a nonzero proportion of invariable sites (WGA + G + I + F), was selected in ProtTest 3.3 (Darriba et al. 2011). Phylogenetic trees were constructed with the neighbor-joining method with 1,000 bootstrap replicates from a matrix of Jones–Taylor–Thornton distances in MEGA5 (Tamura et al. 2011) and using model-based Bayesian inference in MrBayes 3.2.2 (Ronquist and Huelsenbeck 2003). The Bayesian analysis was run for 10^6^ steps with trees sampled every 200 steps and the first 25% of sampled trees discarded as burn-in; two analyses run in parallel, each consisting of one cold and three heated chains, converged on the same posterior distribution as evidenced by the standard deviation (SD) of split frequencies less than 0.01. An additional phylogenetic analysis was performed for the *TLR05* family, which includes members lacking TIR and transmembrane domains; these genes in fish encode a soluble TLR05S form (Tsujita et al. 2004; Palti 2011). Alignment, model selection (also resulting in the WGA + G + I + F model) and phylogenetic analyses were performed as described above. Protein domains were identified using SMART (Letunic et al. 2012).

The selection landscape has been reported to differ between “cell surface” versus “endosomal” (Barreiro et al. 2009) or “viral” versus “nonviral” (Wlasiuk and Nachman 2010) TLRs. To test this hypothesis in newts, we had to modify the criteria of classification because not all newt TLRs could be unambiguously assigned to defined classes: protein (profilin) sensing TLR12 localizes into the endosomal compartment (Yarovinsky 2014), endosomal TLR13 senses bacterial rRNA (Oldenburg et al. 2012) while dsRNA-sensing TLR22 is present on the cell surface (Matsuo et al. 2008). As the core of the hypothesis is the difference in patterns of selection between NA sensing and other TLRs, we classify newt TLRs into two groups according to their ligands: NA-sensing (NA: *TLR03*, *07*, *08*, *09*, *13*, *21*, *22*) or nonNA-sensing TLRs (nonNA: *TLR01*, *02*, *05*, *05 L*, *12*, *14*) (Palti 2011; Oldenburg et al. 2012; Keestra et al. 2013; Koblansky et al. 2013; Pietretti and Wiegertjes 2014; Rauta et al. 2014). The ligand for TLR14 remains unknown, but this gene falls into the *TLR01/02* family and therefore was tentatively classified as nonNA. The *TLR19* ligand is also unknown, but this gene is so divergent from other *TLR* families that we did not assign NA/nonNA status.

### *TLR* Expression in Liver and Spleen

TLRs were identified from deep-sequenced transcriptomes of two organs: liver and spleen. Although only two spleen transcriptomes were available, it was nevertheless possible to meaningfully compare the level of expression of various TLRs between organs. Analysis was performed using a pipeline distributed within the Trinity package. First, it uses RSEM (Li and Dewey 2011) to calculate transcript abundance based on reads mapped to TLRs with Bowtie (Langmead et al. 2009). Next, differentially expressed genes were identified with edgeR (Robinson et al. 2010), a Bioconductor package (Gentleman et al. 2004), with trimmed mean of *M*-values normalization, which accounts for differences between samples in the number of reads. We used a 10^−^^3^ false discovery rate and 4-fold change threshold to identify tissue-biased genes.

### Polymorphism and Divergence of newt *TLR* Genes

Examination of *TLR* polymorphism and divergence within and between closely related species was facilitated by the genomic structure of the vertebrate *TLR* genes: in most genes the entire or an overwhelming majority of the coding region is composed of a single exon. We could therefore design primers for amplification of *TLR* genes from genomic DNA in overlapping 800–1,000 bp fragments (supplementary table S1 and fig. S1, Supplementary Material online). Each polymerase chain reaction (PCR) was performed in 15 µl and contained 7.5 µl of HotStarTaq PCR Master Mix (Qiagen), 1.0 µM of each primer and 50–100 ng of genomic DNA. The following PCR protocol was used: 95°C/15 min, 35 × (94°C/30 s, 55°C/30 s, 72°C/60 s), 72°C/10 min. Amplified fragments were pooled in an equimolar fashion. For each newt, a fragment library was prepared using the Nextera XT kit (Illumina) according to the manufacturer’s protocol. Indexed libraries were sequenced on a MiSeq (Illumina), producing 2 × 150 bp reads. Mapping of reads to reference, single nucleotide polymorphism-calling and physical reconstruction of haplotypes were done as described in Zieliński et al. (2014), resulting in phase-resolved haplotypes (alleles) for most heterozygous individuals. In some cases certain positions could not be physically phased; these were phased computationally using PHASE (Stephens et al. 2001; Stephens and Donnelly 2003); physically phased alleles and homozygous individuals were provided as known haplotypes.

To test the mode of natural selection operating on newt TLR genes, we performed analyses of polymorphism and divergence using two complementary sampling schemes (supplementary fig. S2, Supplementary Material online). A single *L. helveticus* individual was used as an outgroup. In the first scheme 16 *L. montandoni* and *L. vulgaris* individuals were selected, representative of genetic diversity of both species (Babik et al. 2005; Nadachowska and Babik 2009; Nadachowska-Brzyska et al. 2012). This species-wide data set was useful for some selection tests such as the McDonald–Kreitman (MK) test, because high overall variation increases power. Due to strong genetic structure in *Lissotriton*, this sampling strategy was not well suited for neutrality tests based on site-frequency spectra (Städler et al. 2009). Therefore we also used a population sampling strategy, involving two genetically distinct groups which we consider as regional populations: 1) *L. vulgaris graecus* from Albania, Greece and Macedonia and 2) *L. montandoni* from the southern part of its range. Genetic variation within these regional populations is substantial but differentiation between demes is limited (Pabijan et al. forthcoming; Zieliński et al. 2013). Newts form discrete demes corresponding to breeding ponds, such demes undergo extinction and recolonization, and thus the regional population can be considered a metapopulation (Marsh and Trenham 2001; Smith and Green 2005). It has been shown (Wakeley 1999, 2004; Wakeley and Aliacar 2001) that if one gene copy per locus is sampled per deme, the ancestral process producing such sample is identical to the unstructured coalescent process, if time is rescaled appropriately. We approximated this optimal strategy by sampling multiple localities, with one individual sampled per locality. For the species-wide data set we sequenced almost entire coding sequences of *TLR* genes (on average 2.4 kb per gene); for the population data set we focused on the more variable external domain (∼1.5 kb).

Basic measures of polymorphism and divergence including the number of segregating sites (*S*), number of alleles, overall (π), synonymous (π_S_) and nonsynonymous (π_N_) nucleotide diversities, Tajima’s *D* and Fay and Wu’s *H* were calculated in DnaSP 5.1 (Librado and Rozas 2009) and mstatspop (http://bioinformatics.cragenomica.es/numgenomics/people/sebas/software/software.html, last accessed December 20, 2014). For completeness, we report values of *D* and *H* for synonymous, nonsynonymous and all sites combined, but test significance only for synonymous and nonsynonymous sites. Statistical significance of *D* and *H* was tested by comparing the observed values to the null distributions obtained through 100,000 coalescent simulations in ms (Hudson 2002) under an appropriate demographic model (supplementary tables S2 and S3, Supplementary Material online). The demographic model was selected and its parameters estimated using sequences of 62 markers located in 3′-untranslated regions (UTR) of transcripts (Zieliński et al. 2014) within the Approximate Bayesian Computation (ABC) framework; details are given in supplementary material, Supplementary Material online. The null distributions of *D* and *H* for each *TLR* were obtained using synonymous and nonsynonymous mutation rates estimated by comparison with *L. helveticus* which diverged approximately 18.4 Ma (Pabijan et al. forthcoming) and a single recombination rate estimated in the ABC analysis (supplementary table S3, Supplementary Material online).

### Modes and Strength of Natural Selection Acting on newt *TLR* Genes

The maximum-likelihood estimate of the ratio of nonsynonymous to synonymous divergence between *TLR* sequences of *L. vulgaris/L. montandoni* and *Notophthalmus* was calculated in PAML 4.7 (Yang 2007). The effects of nonsynonymous substitutions were predicted using PROVEAN (Choi et al. 2012). SNiPRE (Eilertson et al. 2012), an MK-like method utilizing information from multiple genes to detect selection was used to estimate the measure of constraint *f* (the proportion of nonlethal nonsynonymous mutations). This analysis was done using species-wide data set. The standard MK test was calculated using DnaSP on both species-wide and population data sets.

The Hudson–Kreitman Aguadé (HKA) test assesses heterogeneity of the ratio of intraspecific polymorphism to interspecific divergence; significant heterogeneity indicates that some of the investigated genes have been affected by differential selection. Because HKA is known to be sensitive to population structure (Ingvarsson 2004), we applied this test only to the population data set. The multilocus HKA test was calculated in the program HKA (https://bio.cst.temple.edu/∼hey/software/software.htm); significance was assessed with 10,000 coalescent simulations. Because the HKA test was significant in both lineages, we used the maximum-likelihood version of the test (Wright and Charlesworth 2004) to determine which genes are under selection. Likelihood-ratio tests were used to compare the null neutral model to models in which a particular gene is affected by selection. Tests were done in the program MLHKA (http://wright.eeb.utoronto.ca/programs/) with chain length of 200,000 steps.

The distribution of selection coefficients within the *TLR* genes was investigated using a population genetics-phylogenetics approach implemented in the program gammamap (Wilson et al. 2011). This method is embedded in a Bayesian framework and models evolution of codons to explain both observed intraspecific polymorphism and interspecific divergence. A sliding window approach is used to model variation in selection pressures along genes and infer the posterior probability of positive selection for each codon. The analysis was performed on sequence data from *L. montandoni* and *L. v. graecus*, and a single outgroup (*L. helveticus*) sequence. Prior distributions on all model parameters were chosen as in Wilson et al. (2011). We assumed lineage-specific priors for the distribution of fitness effects (DFE), sliding window smoothing parameter (*p*), branch length (*T*), and transition:transversion ratio (*κ*). An uninformative, symmetric Dirichelet prior was applied on DFE, where 12 fitness classes ranging from effectively inviable to beneficial (γ = −500, −100, −50, −10, −5, −1, 0, 1, 5, 10, 50, 100) are equally likely. We employed a uniform prior on *p* within interval (0, 1), and improper log-uniform priors on *κ* and *T*. The population-scaled mutation parameter θ was assigned a separate log-normal prior distribution for each lineage and each gene with hyperparameters mean μ and SD σ. The mean μ was given an improper uniform prior and SD σ was given a log-normal prior with mean 0 and SD 2. The posterior distributions of selection coefficients for each codon (γ) together with all other parameters were estimated jointly in six separate MCMC runs, each 3,000,000 steps long, with values recorded every 50 steps. Mixing conditions for each parameter were adjusted beforehand. We removed the first 300,000 steps as burn-in and compared chains for convergence. The final estimates were obtained by merging values from all chains together.

## Results

### *TLR* Repertoire and Expression in newts

**Fig. 1.—:**
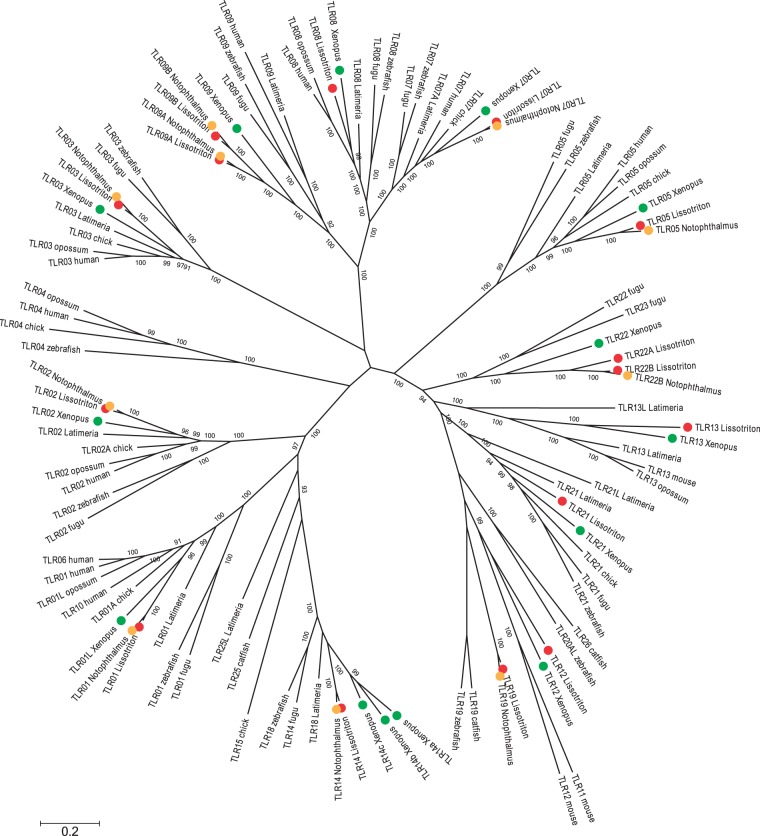
A Bayesian tree showing the relationships among vertebrate TLRs. Amphibian TLRs are color coded by species.

Sixteen *TLR* genes were identified in the *Lissotriton* transcriptome (fig. 1). In most cases assignment to TLR families was straightforward. However, the classification of two genes (*TLR12* and *TLR19*) should be regarded as tentative because of high sequence divergence from other vertebrate genes (fig. 1). Representatives of all major vertebrate *TLR* families except *TLR04* were found in the *Lissotriton* transcriptome. The *TLR* repertoire is similar to that of *Xenopus*. Notable differences include the lack of divergent *TLR14* paralogs, and the presence of *TLR19* which has so far been detected only in fish. *Xenopus* has only four genes in the *TLR11* (sensu Roach et al. 2005) family, whereas *Lissotriton* has six (fig. 1). *TLR09* and *TLR22* underwent duplication in urodeles before the divergence of *Lissotriton* and *Notophthalmus* approximately 70 Ma. Full-length ORFs of 11 and partial ORFs of four apparent orthologs were identified in the *Notophthalmus* transcriptome, making *TLR12* the only *TLR* gene not detected in the available *Notophthalmus* assembly (table 1). A short gene from the *TLR05* family (*TLR05L*), lacking the transmembrane and TIR domains, is present in the newt transcriptome. Phylogenetic analysis demonstrated that it is not orthologous to the teleost soluble *TLR05S* (supplementary fig. S3, Supplementary Material online).

**Table 1:** Synonymous and Nonsynonymous Divergence between *Notophthalmus* and *Lissotriton TLR* Genes

Gene	ncodons	cds	dN	dS	dN/dS
*TLR01*	786	1.00	0.056	0.142	0.39
*TLR02*	799	1.00	0.069	0.184	0.37
*TLR03*	901	1.00	0.057	0.186	0.31
*TLR05*	888	1.00	0.089	0.170	0.52
*TLR05L*	664	1.00	0.064	0.162	0.40
*TLR07*	1,041	0.99	0.048	0.211	0.22
*TLR08*	534	0.50	0.076	0.169	0.45
*TLR09A*	1,038	1.00	0.056	0.154	0.37
*TLR09B*	1,034	1.00	0.079	0.147	0.54
*TLR12*	0	0.00	—	—	—
*TLR13*	566	0.59	0.114	0.160	0.71
*TLR14*	819	1.00	0.050	0.167	0.30
*TLR19*	955	1.00	0.084	0.204	0.41
*TLR21*	707	0.73	0.083	0.316	0.26
*TLR22A*	871	0.91	0.067	0.146	0.46
*TLR22B*	956	1.00	0.059	0.151	0.39

Note.—ncodons, the length of the *Lissotriton-Nothophthalmus* alignment in codons; cds, proportion of the coding sequence covered by alignment; dN, maximum likelihood (ML) estimate of nonsynonymous divergence; dS, ML estimate of synonymous divergence.

**Fig. 2.—:**
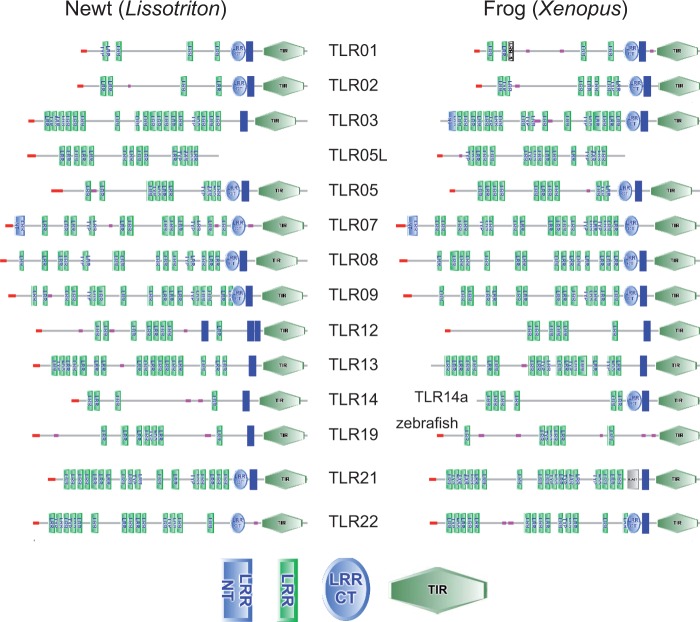
Comparison of the domain structure between newt and frog TLRs. TLR19 is absent in *Xenopus* and zebrafish structure is provided instead; abbreviations of protein domains: LRR NT, Leucine-rich repeat, N-terminal; LRR, Leucine-rich repeat; LRR CT, LRR C-terminal.

Domain structure of newt TLR proteins is generally similar to that of *Xenopus* (fig. 2). Remarkably, neither newt nor frog has a SMART-identifiable transmembrane domain in TLR07. Newts do not have a transmembrane domain in TLR22, a feature shared with fugu TLR22 and 23 proteins but not with *Xenopus* TLR22. The presence of three transmembrane domains (fig. 2) is a unique feature of newt TLR12.

All *TLR* genes were expressed in both liver and spleen although expression level differed widely across genes and organs; the differences spanned almost three orders of magnitude (supplementary fig. S4, Supplementary Material online). Significant differences between organs were detected for five *TLR* genes: *05*, *05L*, and *22A* had higher expression in liver, whereas *TLR12* and *19* had higher expression in spleen (supplementary fig. S4, Supplementary Material online).

### Variation and the Nature of Selection Acting on *TLR* in Lissotriton newts

The presence of more than two expressed variants per individual and high within-individual variation indicate that five genes (*TLR05*, *08 12*, *14*, and *19*) have recently been duplicated in the *Lissotriton* lineage (data not shown). The duplicated copies did not assemble into separate contigs, presumably because of high sequence similarity. We were thus unable to sort out the paralogs and we do not present an analysis of polymorphism for these genes, but use the assembled transcript sequences to assess long-term evolutionary constraint.

The extent of constraint was estimated at three evolutionary scales: 1) long-term, by calculating *Lissotriton*–*Notophthalmus* dN/dS, 2) medium-term, by using *L. montandoni/vulgaris* polymorphism data in relation to divergence from *L. helveticus*, and 3) recent, by employing population genetic and phylogenetic methods in two regional newt populations and controlling for the effects of historical demography. The dN/dS ranged from 0.22 to 0.52, with *TLR13* (for which <60% coding sequence was available for dN/dS calculation) a clear outlier at 0.71 (table 1). No difference in dN/dS was detected between the NA and nonNA TLRs (Welch *t*-test, *t*_10.5_ = 0.4022, *P* = 0.70, *TLR13* excluded). At a medium-term evolutionary scale the range of constraint (*f*, proportion of nonlethal nonsynonymous mutations as calculated by SNiPRE) was relatively narrow (table 2) and did not differ between the NA and nonNA TLRs (*t*_4.9_ = 1.2246, *P* = 0.28). A positive correlation between dN/dS and *f* indicates that the overall constraint has been similar across the timescales (*r* = 0.765, *t*_8_ = 3.3605, *P* = 0.0099).

**Table 2:** Polymorphism in 11 *TLR* Genes in a Species-Wide Sample of *Lissotriton montandoni/vulgaris*, Data for 62 UTR Are Also Shown

Gene	2*N*	nsites	cds	n alleles	*S*	π	π^N^	π^S^	NI	MK *P*-val	*f* Lo95	*f*	*f* Hi95
*TLR01*	32	2,358	1.00	25	160	0.0146	0.0105	0.0293	0.93	1.000	0.23	0.28	0.35
*TLR02*	32	2,313	0.96	21	87	0.0090	0.0066	0.0174	0.74	0.451	0.23	0.30	0.41
*TLR03*	32	1,776	0.66	26	132	0.0136	0.0074	0.0351	0.46	0.086	0.17	0.22	0.27
*TLR05L*	32	1,782	0.89	24	146	0.0117	0.0068	0.0271	0.28	0.063	0.19	0.25	0.30
*TLR07*	32	3,045	0.97	27	171	0.0109	0.0044	0.0331	0.69	0.348	0.16	0.21	0.25
*TLR09A*	32	3,090	0.99	23	118	0.0080	0.0047	0.0194	0.96	1.000	0.20	0.26	0.34
*TLR09B*	30	2,083	0.67	19	119	0.0121	0.0089	0.023	0.40	0.075	0.25	0.31	0.40
*TLR13*	32	2,502	0.88	26	181	0.0100	0.0067	0.0209	1.68	0.303	0.22	0.27	0.33
*TLR21*	32	2,841	0.98	26	215	0.0141	0.0077	0.0347	0.99	1.000	0.18	0.23	0.28
*TLR22A*	32	2,787	0.98	23	149	0.0097	0.0054	0.0242	0.95	1.000	0.20	0.25	0.30
*TLR22B*	32	2,026	0.71	26	145	0.0141	0.0084	0.0334	1.12	1.000	0.22	0.28	0.36
*TLR* average	32	2,418	0.88	24.2	147.5	0.0116	0.0070	0.0271	0.83			0.26	
62 UTR average	32	499		17.7	37.8	0.0146							

Note.—2*N*, number of gene copies sampled; nsites, the length of alignment; cds, proportion of the coding sequence covered by alignment; *S*, number of segregating sites; π, overall nucleotide diversity; π_N_, nucleotide diversity at nonsynonymous sites; π_S_ nucleotide diversity at synonymous sites; NI, neutrality index; MK *P*-val, McDonald and Kreitman Fisher’s exact test *P*-value (*Lissotriton helveticus* was used as an outgroup); *f* Lo95, lower credible limit for constraint calculated in SNiPRE; *f*, estimate of constraint; *f* Hi 95%, upper credible limit for constraint.

Below, we present analyses of polymorphism and divergence based both on species-wide and population data sets (for *L. montandoni* and *L. v. graecus*). *TLR* genes exhibited comparable variation in the species-wide data set and their average π was about 0.8 of that for 62 3′-UTRs (table 2 and supplementary fig. S5, Supplementary Material online). No differences in nucleotide diversity between the NA and nonNA TLRs were detected (*t*_3.063_ = 0.113, *P* = 0.917).

The MK test did not detect adaptive evolution for any *TLR* in the species-wide data set (table 2), but in population data sets significant results were obtained for *TLR05L* in *L. montandoni* (*P* = 0.035) and *TLR09B* in *L. v. graecus* (*P* = 0.006), indicating that either power was increased in relation to the species-wide data set or that selection pressures have varied in the history of the two evolutionary lineages. The multilocus HKA test was significant in both lineages (*L. montandoni P* < 0.0001, *L. v. graecus P* = 0.007). In individual tests two genes exhibited an excess of divergence: *TLR05L* in *L. montandoni* (*P* = 0.006) and *TLR21* in *L. v. graecus* (*P* = 0.001) and one gene, *TLR07*, showed a marginally significant excess of polymorphism (*P* = 0.04) in *L. v. graecus* (table 3).

**Table 3:** Polymorphism in 11 *TLR* Genes in Population-Level Samples of Two Evolutionary Lineages: *Lissotriton montandoni* (*Lm*, 38 gene copies sampled) and *L. vulgaris graecus* (*Lvg*, 34 gene copies sampled), Data for 62 UTR Are Also Shown

Gene	nsites	cds	n alleles		*S*		π		π_S_		π_N_	
			*Lm*	*Lvg*	*Lm*	*Lvg*	*Lm*	*Lvg*	*Lm*	*Lvg*	*Lm*	*Lvg*
*TLR01*	1,626	0.69	13	18	65	39	0.0112	0.0047	0.0220	0.0113	0.0081	0.0028
*TLR02*	1,656	0.69	10	12	23	35	0.0024	0.0031	0.0074	0.0047	0.0009	0.0027
*TLR03*	1,620	0.60	17	19	65	47	0.0097	0.0037	0.0289	0.0079	0.0041	0.0025
*TLR05L*	1,463	0.73	5	18	5	41	0.0008	0.0051	0.0019	0.0139	0.0004	0.0024
*TLR07*	1,527	0.49	22	17	44	58	0.0067	0.0065	0.0143	0.0195	0.0045	0.0026
*TLR09A*	1,683	0.54	7	14	27	39	0.0056	0.0048	0.0097	0.0112	0.0034	0.0029
*TLR09B*	1,452	0.47	14	16	15	26	0.0017	0.0046	0.0029	0.0105	0.0013	0.0028
*TLR13*	1,479	0.52	16	14	46	48	0.0092	0.0047	0.0168	0.0067	0.0068	0.0040
*TLR21*	1,611	0.55	25	11	82	15	0.0124	0.0017	0.0283	0.0033	0.0074	0.0015
*TLR22A*	1,723	0.60	9	16	47	47	0.0095	0.0041	0.0221	0.0130	0.0058	0.0015
*TLR22B*	1,387	0.48	18	18	53	43	0.0095	0.0070	0.0219	0.0151	0.0058	0.0047
*TLR* average	1,566	0.58	14.2	15.7	42.9	39.8	0.0071	0.0045	0.0160	0.0106	0.0044	0.0028
62 UTR average	499		7	9.5			0.0044	0.0052				

Note.—nsites, the length of alignment; cds, proportion of the coding sequence covered by alignment; *S*, number of segregating sites; π, overall nucleotide diversity; π_N_, nucleotide diversity at nonsynonymous sites; π_S_ nucleotide diversity at synonymous sites.

**Fig. 3.—:**
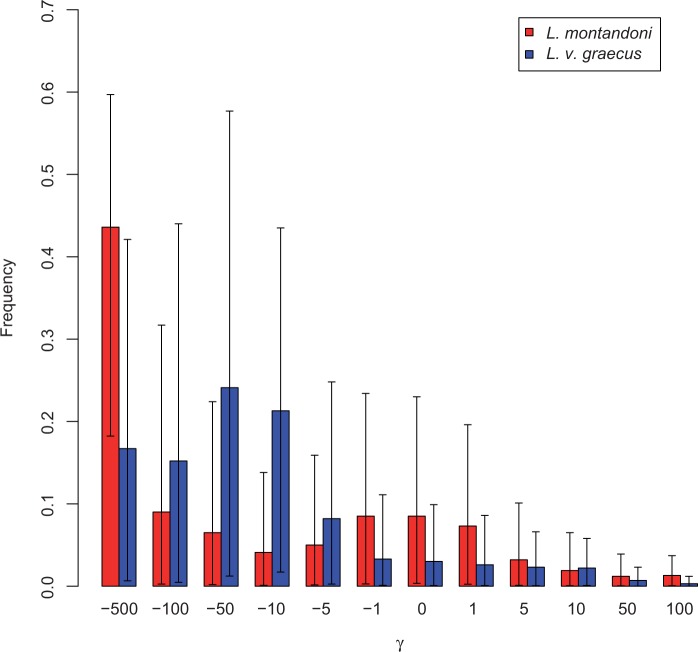
The distribution of fitness effects of new mutations in 11 *Lissotriton TLR* genes.

The distribution of selection coefficients for 11 *TLR* genes as estimated by the population genetics-phylogenetics gammamap method differed substantially between *L. montandoni* and *L. v. graecus*, although the 95% credible intervals estimated for each fitness class were wide in both species (fig. 3). The proportion of strongly constrained codons (−500 ≤ γ ≤ −5) was higher in *L. v. graecus* than in *L. montandoni* (86% vs. 68%). The frequencies of neutral and beneficial mutations in *L. montandoni* exceeded those in *L. v. graecus*, with the starkest contrast among neutral and nearly neutral mutations (−1 ≤ γ ≤ 1, 24% vs. 9%). Despite overall constraint, adaptive evolution may have occurred at individual codons. Following Quach et al. (2013), we considered codons as evolving under positive selection if the proportion of simulated positive gamma values (γ > 0) exceeded 0.75. In total 33 codons under positive selection were inferred in *L. v. graecus*, compared with only 15 in *L. montandoni* (supplementary table S4, Supplementary Material online). Overall, the number of positively selected codons per gene was low, ranging from zero in *TLR01* in both lineages to 8 in *TLR09B* in *L. v. graecus* and only four were predicted to have a radical effect on protein function (supplementary table S4, Supplementary Material online). In five genes (*TLR02, 09A, 09B, 21, 22B*) positively selected codons were detected in both lineages. In addition, positive selection was inferred in four genes (*TLR03, 07, 13, 22A*) only in *L. v. graecus*, and in one gene (*TLR05L*) in *L. montandoni*. Remarkably, only a single codon was identified as positively selected in both lineages (codon 294 of *TLR21*, in which a nonsynonymous polymorphism shared with the outgroup species segregates in both lineages). Nonsense mutations segregated at appreciable frequencies in *TLR09B* in *L. montandoni*, the more common of these mutations produced a stop codon in position 48, indicating that the respective allele does not encode a functional protein.

**Fig. 4.—:**
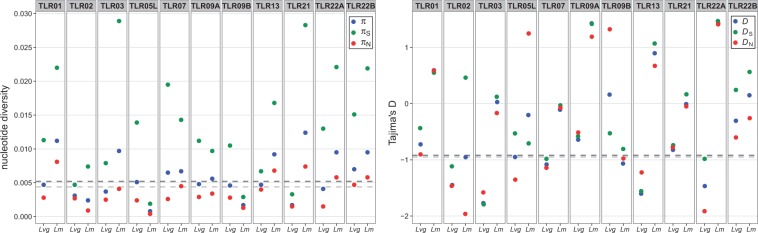
Nucleotide diversity and Tajima’s *D* in population data sets; *Lm*, *Lissotriton montandoni*, *Lvg*, *L. vulgaris graecus*; π, overall, π_S_, synonymous and π_N_, nonsynonymous nucleotide diversity; *D*, overall, *D*_S_, synonymous and *D*_N_, nonsynonymous Tajima’s *D*.

In the population data sets *TLR* nucleotide diversity tended to be higher in *L. montandoni* (π_mon_**= 0.0072, π_gre_ = 0.0045, *t*_12.61_ = 2.027, *P* = 0.064), whereas for 62 3′-UTRs it was similar in both lineages (π_mon_ = 0.0044, π_gre_ = 0.0052, *t*_121.9_ = 1.079, *P* = 0.283) (table 3 and fig. 4). Tajima’s *D* in *TLR* genes was significantly higher in *L. montandoni* (*D*_mon_ = 0.20, *D*_gre_ = −0.96, *t*_18.28_ = −3.827, *P* = 0.0012), whereas no differences were detected in UTRs (*D*_mon_ = −0.95, *D*_gre_ = −0.92, *t*_120.9_ = 0.175, *P* = 0.086) (table 4 and fig. 4). In each lineage Tajima’s *D* values at synonymous and nonsynonymous sites of most genes were remarkably similar and in most cases did not depart from expectations under the inferred demographic model (supplementary tables S2 and S3, Supplementary Material online; table 4 and fig. 4). The few significant *D* values indicated an excess of high frequency variants (table 4). Significantly negative Fay and Wu’s *H*, signaling an excess of high-frequency derived alleles consistent with an incomplete selective sweep was detected in one *L. montandoni* gene (*TLR22B*, only synonymous sites), whereas three (*TLR03, 07, 13*) *L. v. graecus* genes exhibited this pattern (table 4). The same three genes were identified by the gammamap method as targets of positive selection in *L. v. graecus*.

**Table 4:** Results of Neutrality Tests and Values of Tajima’s *D* and Fay and Wu’s *H* Statistics in *Lissotriton montandoni* (*Lm*, 38 gene copies sampled) and *L.vulgaris graecus* (*Lvg*, 34 gene copies sampled)

	MK *P*-val		HKA *P*-val		*D*		*D*_S_		*D*_N_		*H*		*H*_S_		*H*_N_	
	*Lm*	*Lvg*	*Lm*	*Lvg*	*Lm*	*Lvg*	*Lm*	*Lvg*	*Lm*	*Lvg*	*Lm*	*Lvg*	*Lm*	*Lvg*	*Lm*	*Lvg*
*TLR01*	0.806	0.644	0.273	0.968	0.57	**−**0.74	0.52	**−**0.45	0.57	**−**0.94	0.75	**−**0.98	**−**0.11	**−**2.50	1.05	1.63
*TLR02*	1.000	0.621	0.108	0.695	**−**0.93	**−**1.41	0.50	**−**1.08	**−**1.93	**−**1.45	**−**6.15	**−**4.03	**−**3.25	**−**1.46	**−**2.95	**−**3.72
*TLR03*	0.264	0.824	0.471	0.752	0.00	**−**1.79	0.10	**−**1.77	**−**0.19	**−**1.63	4.14	**−**16.66	3.49	**−**11.46*	0.55	**−**5.14
*TLR05L*	0.035	0.170	0.006	0.359	**−**0.16	**−**0.94	**−**0.72	**−**0.52	1.25	**−**1.34	0.66	0.19	0.32	**−**0.55	0.24	0.74
*TLR07*	0.139	0.741	0.152	0.041	**−**0.06	**−**1.07	0.00	**−**0.99	**−**0.10	**−**1.10	2.13	**−**6.45	0.11	**−**3.08	1.97	**−**3.26*
*TLR09A*	0.435	1.000	0.165	0.735	1.38	**−**0.61	1.39*	**−**0.60	1.18	**−**0.56	**−**4.38	2.44	**−**1.46	0.02	**−**1.88	1.98
*TLR09B*	0.112	0.006	0.115	0.305	**−**1.06	0.15	**−**0.84	**−**0.53	**−**1.01	1.29*	0.84	**−**1.81	**−**0.20	**−**1.54	0.76	**−**0.33
*TLR13*	0.361	0.576	0.206	0.234	0.85	**−**1.63	1.04	**−**1.58	0.63	**−**1.20	3.20	**−**11.04	1.01	**−**5.43*	2.19	**−**5.28*
*TLR21*	0.843	0.452	0.594	0.001	0.01	**−**0.83	0.12	**−**0.76	**−**0.07	**−**0.74	**−**5.17	**−**1.78	**−**1.21	**−**0.93	**−**2.20	1.13
*TLR22A*	0.818	0.198	0.685	0.743	1.43	**−**1.45	1.43*	**−**1.01	1.37*	**−**1.92	1.91	**−**7.97	0.94	**−**3.30	0.99	**−**4.04
*TLR22B*	0.723	1.000	0.140	0.123	0.15	**−**0.26	0.58	0.25	**−**0.25	**−**0.62	**−**7.10	**−**0.65	**−**4.23*	2.95	**−**0.89	0.23
*TLR* average					0.20	−0.96	0.37	−0.82	0.13	−0.93	−0.83	−4.43	−0.42	−2.48	−0.01	−1.46

Note.—MK *P*-val, McDonald and Kreitman Fisher’s exact test *P*-value; HKA *P*-val, maximum-likelihood HKA test *P*-value; *D*, Tajima’s *D* value for all sites; *D*_S_, Tajima’s *D* value for synonymous sites; *D*_N_ Tajima’s *D* value for nonsynonymous sites; *H*, Fay and Wu’s *H* value for all sites; *H*_S_, Fay and Wu’s *H* value for synonymous sites; *H*_N_, Fay and Wu’s *H* value for nonsynonymous sites; *, *P* < 0.01; *P*-values are provided only for *D*_S_, *D*_N_, *H*_S_, and *H*_N_, for *D* two-sided, for *H* one-sided.

## Discussion

This work is the first analysis of the repertoire and evolutionary dynamics of *TLR* genes in urodele amphibians and one of the few studies employing population genetic methods to understand processes shaping variation in these genes, while controlling for nonequilibrium demography. Overall, we found that amid substantial evolutionary conservation episodes of lineage-specific adaptation have involved individual codons of some *TLR* genes, potentially providing a more efficient response to pathogen assault that may vary in space and time. Available data from several systems indicate that lineage-specific bouts of adaptive evolution may dominate the dynamics of vertebrate TLRs (Tschirren et al. 2011, 2012; Quach et al. 2013; Grueber et al. 2014). Thus, although adaptive changes in TLR genes may be subtle in comparison to other components of the immune system (Piertney and Oliver 2006; Downing et al. 2010; Quintana-Murci and Clark 2013), these genes should be incorporated into research aiming to understand associations between genetic variation and susceptibility to pathogens in natural populations (Tschirren et al. 2013).

### The Evolution of the Urodele *TLR* Repertoire

Substantial differences in the composition of the *TLR* family occur between fish and amniotes (Roach et al. 2005; Temperley et al. 2008; Palti 2011). Amphibians have been suggested to possess an intermediate *TLR* repertoire which may reflect their association with both aquatic and terrestrial environments (Ishii et al. 2007; Oshiumi et al. 2008). The data so far have been restricted to *Xenopus* (Ishii et al. 2007). Our study shows that *TLR* composition in urodeles and anurans is broadly similar. The only major family present in newts but absent in *Xenopus* is *TLR19*, otherwise found only in some fish (Palti 2011), and newts express representatives of all families present in *Xenopus*. No *TLR04* orthologs have been found in the newt transcriptome, leaving *TLR04* the only major vertebrate *TLR* family not detected in urodeles. TLR04 plays a crucial role in the mammalian response against diverse microbes, especially gram-negative bacteria (Akira et al. 2006). In fish TLR04 is functionally different from the mammalian counterpart and present only in some species (Palti 2011; Kanwal et al. 2014). The presence of *TLR04* in *Xenopus* has been controversial (Roach et al. 2005; Ishii et al. 2007), but *TLR04* genes derived from automated prediction are present in the most recent versions of the *Xenopus laevis* and *Xenopus tropicalis* genome assemblies (http://www.xenbase.org/). *TLR04* has also been reported in the transcriptome of another anuran, *Bombina maxima* (Zhao et al. 2014), and, based on indirect data, also in frogs (Nikolaeva et al. 2012). Thus the evidence regarding the presence and nature of amphibian *TLR04* is somewhat equivocal. A short amphibian gene from the *TLR05* family lacking the TIR domain (*TLR05L*) is not orthologous to the teleost soluble *TLR05S* (Muñoz et al. 2013), but is unique to some tetrapods, being found in turtles (supplementary fig. S3, Supplementary Material online) and the anole lizard (Muñoz et al. 2013). It is currently unknown whether TLR05L binds flagellin and modulates a flagellin-mediated immune response, as has been demonstrated for teleost TLR05S (Muñoz et al. 2013). The expression of newt *TLR05L* was much higher in liver than in spleen, similar to that of fish *TLR05S*.

Lineage-specific duplications have occurred in anurans and urodeles, leading to differences in the composition of several *TLR* families between the amphibian orders. At least 16 *TLR* genes are expressed in newts, four of them result from duplications (*TLR09A-09B* and *TLR22A-22B*) predating the divergence of *Lissotriton* and *Notophthalmus* at approximately 70 Ma (Roelants et al. 2007). Duplications have also been common in the more recent history of the *Lissotriton TLR* genes, as evidenced by closely related paralogs in five of them, which brings the total number of *TLR* genes to at least 21. Due to high sequence similarity, examination of variation in these *TLR* genes would require a separate study employing dedicated methods. Such studies are desirable as they may shed light on the fates of *TLR* paralogs immediately following duplication: whether both copies are commonly maintained by selection or if one typically undergoes pseudogenization. Gene duplication and to a lesser extent, gene conversion among paralogs within families, feature prominently in the evolution of vertebrate *TLR* genes. Mammals and birds differ in this respect from fish. Although in the former duplication and gene conversion are restricted mainly to the *TLR01* family (Huang et al. 2011), in the latter duplications are common, particularly in fish-specific TLRs, resulting in multigene families sometimes composed of many similar paralogs (Star et al. 2011; Sundaram et al. 2012; Quiniou et al. 2013; Pietretti et al. 2014). Duplications in fish seem lineage-specific and short lived, as their patchy phylogenetic distribution suggests. The data from newts reveal a similar pattern with recent duplications in four of five major *TLR* families. The propensity for retention of duplicated *TLR* copies may be related to the efficiency of other components of the immune system as has been suggested in the case of extreme duplications of *TLR* in the Atlantic cod, a fish species which lacks a functional major histocompatibility complex (MHC) class II pathway (Star et al. 2011; Sundaram et al. 2012).

### Constraint and Lineage-Specific Adaptation in newt *TLR* Genes

Urodele *TLR* genes are substantially constrained at the long-term evolutionary scale, and the extent of constraint in individual genes is similar to that reported for birds (Alcaide and Edwards 2011; Grueber et al. 2014). Thus purifying selection has predominated the evolution of urodele *TLR* genes, similarly as in other vertebrates (Roach et al. 2005; Mikami et al. 2012). However, several studies found signatures of recurrent or episodic positive selection affecting a relatively minor (<1–5%) fraction of codons in birds and mammals (Wlasiuk et al. 2009; Wlasiuk and Nachman 2010; Alcaide and Edwards 2011; Areal et al. 2011; Grueber et al. 2014). With data from only two species, we did not have enough power to test for individual codons under positive selection, but flag this as a priority for future studies with more data.

We used the extensive data on polymorphism and divergence of *TLR* genes to test for selection in the more recent evolutionary history of *Lissotriton* newts, that is, since the origin of the *L. vulgaris* lineage at approximately 18 Ma (Pabijan et al. forthcoming). At this scale constraint remains strong; no differences were detected between NA-sensing and nonNA-sensing *TLR* genes. Stronger purifying selection in the former versus more positive selection and less constraint in the latter were reported in primates and birds (Barreiro et al. 2009; Wlasiuk and Nachman 2010; Huang et al. 2011). Conservation of NA-sensing TLRs has been ascribed to functional constraint, that is, proteins encoded by these genes must recognize motives and types of foreign NA without triggering autoimmune reactions. This may well be true for specific clades, but a broader analysis of mammal *TLR* did not confirm differences in the rate of adaptive evolution between viral and nonviral *TLR* genes (Areal et al. 2011). Likewise, we did not find this pattern in newts.

Our results suggest substantial differences in selection regimes between the two closely related lineages *L. montandoni* and *L. v. graecus*; their divergence has been estimated at approximately 7 Ma (supplementary table S3, Supplementary Material online). Natural selection operating on *TLR* genes has been stronger in *L. v. graecus* and, interestingly, both purifying and positive selection seem more prominent in this lineage compared with *L. montandoni* in which selection appears somewhat relaxed. Despite the overwhelming signal of purifying selection, positive selection was detected by at least one method in 9 of 11 genes in *L. v. graecus*, but only in 6 *L. montandoni* genes. Also over twice as many codons evolved adaptively in the former lineage compared with the latter. Recent positive selection in *L. v. graecus* is emphasized by concomitant signals of incomplete sweeps in the Fay and Wu test and positive selection inferred by the gammamap method in three genes. Qualitatively similar differences in overall *TLR* selection landscapes have been reported between humans and other great apes and attributed to differences in demographic history (Quach et al. 2013). It is unlikely that our inference of selection has been severely distorted by demographic factors in newts because effective population sizes and demographic histories of both lineages were similar as estimated from an independent data set using ABC modeling. Therefore the strength of selection may signify differences between lineages in the overall importance of TLRs for an effective immune response. Because MHC class II variation is lower in *L. v. graecus* compared with *L. montandoni* (Nadachowska-Brzyska et al. 2012), it is tempting to speculate that the innate components of the *L. v. graecus* immune system may be relatively more important in fighting pathogen assault. Interestingly, a recent study reported drastic differences between species of the genus *Lissotriton* in susceptibility to an emerging urodele fungal pathogen *Batrachochytrium salamandrivorans* (Martel et al. 2014) which may suggest substantial interspecific immunogenetic differences.

The strongest and most consistent signatures of positive selection were observed in *TLR05L* of *L. montandoni*. This gene exhibits an excess of fixed replacement substitutions, an excess of divergence, and two codons with fixed substitutions identified by gammamap as targets of positive selection. Although direct evidence is lacking, it is likely that TLR05L modulates, together with TLR05, a flagellin-mediated immune response (see above). *TLR05* is a common target of positive selection (Wlasiuk et al. 2009) that may be driven by coevolution with bacteria which, in some bacterial species, leads to variation in flagellin sequences resulting in the evasion of recognition by hosts (Andersen-Nissen et al. 2005). Thus positive selection in *L. montandoni TLR05L* may reflect adaptation to changing pathogen pressure. In this context it would be interesting to investigate *TLR05* variation and test for recent selection in this gene which we were unable to do because of the presence of closely related paralogs.

The MK test and gammamap detected signatures of positive selection acting on *TLR09B* in *L. v. graecus*. TLR09 senses mainly unmethylated CpG-rich bacterial DNA and DNA:RNA hybrids (Bauer et al. 2001; Yeh et al. 2013; Rigby et al. 2014). Although *TLR09* is generally strongly constrained in mammals, a few codons under positive selection have been reported (Wlasiuk and Nachman 2010; Areal et al. 2011) and adaptive evolution of this gene may be more frequent in fish (Chen et al. 2008; Zhu et al. 2013). The importance of *TLR09* in fish antibacterial immunity is suggested by two observations. First, in zebrafish two distantly related proteins, TLR09 and TLR21, mediate CpG oligonucleotide activity, but have different recognition profiles (Yeh et al. 2013). Second, the only species in which *TLR09* duplication has been reported so far is cod, which lacks MHC class II signaling but has five *TLR09* paralogs; expansion of the *TLR09* family may reflect its importance for antibacterial immunity in this species (Star et al. 2011). In contrast to birds and mammals with either *TLR09* or *TLR21*, amphibians have both receptors, thus the role of these receptors in antibacterial response may be similar to that of fish. Because CpG DNA from various bacteria differ in their potential to activate TLR09 (Bauer et al. 2001), coevolution between host TLR and bacterial pathogens appears likely. The two urodele *TLR09* paralogs have been maintained by selection for at least tens of millions of years, so possibly these genes specialized in recognition of various bacterial DNA PAMPs. Contrary to the situation observed in *L. v. graecus*, relaxation of selection and some functional redundancy of *L. montandoni TLR09B* is suggested by the relatively high frequency (∼15%) of a nonsense (premature stop codon) mutation. Patterns consistent with recent positive selection were also detected in *TLR21* of *L. v. graecus*: an excess of divergence, low variation, and six replacement substitutions fixed by selection as inferred by gammamap. Again, relaxation of selection in *L. montandoni* is more likely: *TLR21* is the most variable of all *TLR* genes and nonsynonymous polymorphisms are still segregating in all codons identified as positively selected by gammamap. Hence, *L. v. graecus* and *L. montandoni* likely differ in the response to bacterial DNA mediated by TLR09 and TLR21 and such differences may reflect variable selection pressure exerted by pathogens on *TLR* genes in closely related evolutionary lineages.

## Conclusions

In this study, we characterized urodele *TLR* genes which encode a crucial component of the innate immune system and provide a link to adaptive immunity. The *TLR* repertoires of two major amphibian groups, urodeles and anurans, are broadly similar and intermediate between those of fish and amniotes. Gene duplications feature prominently in the evolution of urodele *TLR*. Both old duplications, apparently maintained by long-term selection and very recent duplications are present. *TLR* genes examined for polymorphism and divergence in *Lissotriton* newts exhibit a comparable degree of conservation and evolutionary constraint. However, the rate and targets of adaptive evolution differ substantially between two recently diverged *Lissotriton* lineages. These differences were not an artifact of nonequilibrium demography because demographic effects were accounted for through ABC modeling using an independent data set of 62 loci. Short-term selective pressures thus vary both between closely related lineages and among *TLR* genes, and may indicate differences in the relative roles of innate and acquired immunity. *TLR* variation may play an important role in the response to changing pathogen pressure, and these genes are a promising target for studies aiming to link genetic variation to pathogen susceptibility in amphibians, a vertebrate group severely threatened by emerging infectious diseases.

## Supplementary Material

Supplementary material, tables S1–S4, and figures S1–S5 are available at *Genome Biology and Evolution* online (http://www.gbe.oxfordjournals.org/).

Supplementary Data
