# Supplementary material for: Constraint and Adaptation in newt Toll-Like Receptor Genes
Source: Genome Biol Evol. 2014 Dec 4;7(1):81–95. doi: 10.1093/gbe/evu266 (PMC4316619; doi:10.1093/gbe/evu266)
Supplement: Supplementary Data [file supp_7_1_81__index.html]

Constraint and adaptation in newt Toll-like receptor genes — Constraint and Adaptation in newt Toll-Like Receptor Genes — Supplementary Data 

# Constraint and Adaptation in newt Toll-Like Receptor Genes

## Supplementary Data

files

**Files in this Data Supplement:**

- Supplementary Data - zip file
