# Supplementary material for: Constraint and Adaptation in newt Toll-Like Receptor Genes
Source: Genome Biol Evol. 2014 Dec 4;7(1):81–95. doi: 10.1093/gbe/evu266 (PMC4316619; doi:10.1093/gbe/evu266)
Supplement: Supplementary Data [file supp_evu266_suppl_data.zip › Babik_et_al_Supplementary_Information_without_code_fields_revision.doc]

**Supplementary Information for the article**

**Babik et al. Constraint and adaptation in newt Toll-like receptor genes.**

**Supplementary Methods**

*Historical demography*

Tests of selection based on the frequency spectra are sensitive to nonequilibrium demography (Nielsen 2005). Therefore null distributions of test statistics under a demographic model adequately describing the history of investigated populations are needed to adequately control the type I error. We used sequences of 62 3’UTR fragments (Zieliński et al. 2014) to evaluate several simple demographic models for *L. montandoni* and *L. vulgaris graecus* and to estimate parameters of the best-fitting model. Model selection and parameter estimation were performed within the Approximate Bayesian Computation (ABC) framework (Beaumont 2010; Bertorelle et al. 2010). The model was then used to obtain null distributions of Tajima’s *D* and Fay & Wu’s *H* statistics.

*Models*

Eight models describing the historical demography and divergence of *L. montandoni* and *L. v. graecus* were evaluated. We kept the models relatively simple to obtain reliable estimates of parameters from the available data, but even such simple models should be able to capture the main features of the historical demography. These models were:

i) M1 – a simple model of population divergence without gene flow and constant descendant population sizes,

ii) M1GF – constant descendant population sizes with constant asymmetric migration between populations,

iii) M1GFA – constant descendant population sizes with old asymmetric migration between population,

iv) M2 – a single, instantaneous size change within each descendant population after split but no gene flow; two versions of this model were evaluated: with a narrow (M2N) and wide (M2W) prior for the time of the demographic change,

v) M2GF – a combination of models M1GF and M2 with instantaneous size change within descendant populations and constant asymmetric gene flow; two versions of this model were evaluated: with a narrow (M2GFN) and wide (M2GFW) prior for the time of the demographic change,

vi) M2GFA – a single, instantaneous size change within each descendant population after split and ancestral gene flow.

*ABC methodology*

We analyzed the data using the ABC framework as implemented in ABCtoolbox 1.5 (Wegmann et al. 2010; D. Wegmann kindly provided a new version). Simulations were performed with fastsimcoal21 (Excoffier and Foll 2011). The following summary statistics were used: the overall Tajima’s *D*, nucleotide diversity, number of segregating sites, number of fixed differences, number of shared polymorphisms, number of segregating sites private to *L. montandoni*, number of segregating sites private to *L. v. graecus*, *F*_ST_ and within lineage Tajima’s *D* and nucleotide diversity. However due to high correlation between some of these statistics, the overall Tajima’s *D*, overall nucleotide diversity and within lineage nucleotide diversities were excluded from inference. The data were subsampled to the lowest number of observations per locus (15 individuals for *L. montandoni* and 13 individuals for *L. v. graecus*) and gaps were excluded before observed summary statistics were calculated. The final dataset consisted of 62 loci of average length 479 bp. Summary statistics were calculated in mstatspop v.0.998980beta (Ramos-Onsins et al. unpublished, http://bioinformatics.cragenomica.es/numgenomics/people/sebas/software/software.html ).

*Priors, model choice and posteriors*

For most parameters prior ranges were based on the results of previous or ongoing studies (Nadachowska and Babik 2009; M. Stuglik, W. Babik unpublished; P. Zieliński, W. Babik upublished) but we ensured that prior ranges were wide enough to cover the range of plausible parameter values. We used uniform prior distributions for all parameters except population sizes which were sampled from distributions uniform on a log10 scale. For each model we ran 10^5^ exploratory simulations to examine whether the model was able to explain the observed data; we inspected posterior probability curves to check if models could be improved by changing ranges of prior distributions. Prior ranges were updated, if necessary, for all models simultaneously. Finally one million datasets were simulated under each demographic model. We retained 1% (10^4^) best simulations for each model and computed the likelihood of the observed and retained datasets under GLM (Leuenberger and Wegmann 2010). The reported *P*-value is the fraction of the retained simulations with likelihood lower or equal to the likelihood of the observed data. The best fitting model was selected via Bayes factors (ratios of model marginal densities).

**Supplementary results**

*Demographic history inferred by ABC*

The observed summary statistics fell well within the density distribution of the retained simulations for M1GF (0.34), M1GFA (0.76), M2GFW (0.52), M2GFN (0.83), and M2GFA (0.31), i.e. all models which included gene flow (Table S2). Model M2GFA, which assumes old gene flow and allows for a demographic change in each descendant population had the highest posterior probability (PP = 0.92). Posterior distributions of the model parameters are characterized in Table S3.

**Supplementary references**

Beaumont MA. 2010. Approximate Bayesian computation in evolution and ecology. Ann Rev Ecol Evol Syst. 41: 379-406.

Bertorelle G, Benazzo A, Mona S. 2010. ABC as a flexible framework to estimate demography over space and time: Some cons, many pros. Mol Ecol. 19: 2609-2625.

Excoffier L, Foll M. 2011. fastsimcoal: A continuous-time coalescent simulator of genomic diversity under arbitrarily complex evolutionary scenarios. Bioinformatics 27: 1332-1334.

Leuenberger C, Wegmann D. 2010. Bayesian computation and model selection without likelihoods. Genetics 184: 243-252.

Nadachowska K, Babik W. 2009. Divergence in the face of gene flow: the case of two newts (Amphibia: Salamandridae). Mol Biol Evol. 26: 829-841.

Nielsen R. 2005. Molecular signatures of natural selection. Ann Rev Genet. 39: 197-218.

Wegmann D, Leuenberger C, Neuenschwander S, Excoffier L. 2010. ABCtoolbox: A versatile toolkit for approximate Bayesian computations. BMC Bioinformatics 11: 116.

Zieliński P, Stuglik MT, Dudek K, Konczal M, Babik W. 2014. Development, validation and high-throughput analysis of sequence markers in nonmodel species. Mol Ecol Res. 14: 352-360.

Table S1. Primers used in the study

| TLR gene | primer name | sequence 5'->3' |
| --- | --- | --- |
| TLR01 | TLR01_1F_2 | GACCCACAAGCATCTTCAAA |
|  | TLR01_1R | TTACTCCAGCTCAATGTCACAT |
|  | TLR01_2F | AAGTTTGAGGCTACACACTGT |
|  | TLR01_2R | TGGTTACTGTGCAGATCAAGC |
|  | TLR01_3F | AGTAAGCTCTATGACCACCCA |
|  | TLR01_3R | AGACCCATCTTCCCTCTCCA |
|  | TLR01_4F | ACCTTGTCTTTGTTTTGCCTGT |
|  | TLR01_4R | GCATCCATTTCCAACCACTCA |
|  | TLR01_1R2 | AGAAGGCCTGAAACAACTTAACA |
|  | TLR01_2F2 | AAAGGAYATGGACCCCTAC |
|  | TLR01_2R2 | CATYGGTAAGACTCAGGCCA |
| TLR02 | TLR02_1F_2 | CCCGTTCAGAGAACCCTACA |
|  | TLR02_1R | CTAATCGAGCCTGGCCCAG |
|  | TLR02_2F | TGGCCATAGAAAATCATCCCTT |
|  | TLR02_2R | GTGTTTTAGGCCAGTGACACA |
|  | TLR02_3F | TGCTTTTCGATTTGGCTTTGA |
|  | TLR02_3R | CTTCTTGGTTGTCTCTTTGCCT |
|  | TLR02_4F | TGGCCAAAGGACTATGTATGTG |
|  | TLR02_4R_2 | TTGGTCCCTCTAAATTCTTAGCA |
|  | TLR02_1F3 | TGGTCCWTGTGCATCGTTKT |
|  | TLR02_1R3 | CCCACACCATGACAGAAGGA |
|  | TLR02_2F3 | GTCCMRAGAGTGTAGCAGGA |
|  | TLR02_2R4 | TTAACGTCTGATGGCACCAG |
|  | TLR02_2F2 | AGCAGGAATGGATGTCTTGAT |
|  | TLR02_2R2 | TCCTCAACTTGAATTCCCCTCA |
| TLR03 | TLR03_1F | GTTCCAGGCTGTTTTCATGC |
|  | TLR03_1R | TGGGGACGGACTTAGATTCA |
|  | TLR03_2F | TCAATCTCACGGAAAGTGGA |
|  | TLR03_2R | CTGTCTTTGCATGGAGCATT |
|  | TLR03_3F | GCTTTCAAGGGCTTGTCTCA |
|  | TLR03_3R | ACCTTTCTGCTGCTTTGCAT |
|  | TLR03_1R2 | CCAGGATTTGCVCTCTTCC |
|  | TLR03_2F2 | ACGYCTCATATTGACAAGTGA |
|  | TLR03_2R2 | TGTCTACCCAKTCAGTGTCCT |
| TLR05L | TLR05L_1F | ACTGTACCATGTCTTGCTGGA |
|  | TLR05L_1R | GCTGCATTGACATCCGGWAT |
|  | TLR05L_2F | ACCAATGTGTACCAACCCCT |
|  | TLR05L_2R | TGTACCCAGSGAGTTGTGG |
|  | TLR05L_3F | ATGGCATASGTTCGTTTGCC |
|  | TLR05L_3R | TCCAACAGACCACAGTGACA |

Table S1 continued

| TLR gene | primer name | sequence 5'->3' |
| --- | --- | --- |
| TLR07 | TLR07_1F | GCTGTACTCAACTATCCTGCG |
|  | TLR07_1R | TGTGAATCTCAATTGCGGCA |
|  | TLR07_2F | TCCCTGCAACGTGTCATTTT |
|  | TLR07_2R | AATGTGTGTCCTGAAGCCGT |
|  | TLR07_3F | TTAGACCACTGATCCCGCTC |
|  | TLR07_3R | TGCCTTCAAAACTCCCTGGA |
|  | TLR07_4F | GCCCTCTCTGAAGAAACTGC |
|  | TLR07_4R | CCTTTTAATTTGGCCGCACAG |
|  | TLR07_5F | AGCAGTATGGTTTGTGTGGTG |
|  | TLR07_5R | GTTGTGAGTCGTTATGCATGGA |
|  | TLR07_1R3 | TGGCCTCYYCCATTTCATGT |
|  | TLR07_2F2 | GAAAATTTTGCGTCTTAGGAGCA |
|  | TLR07_2R2 | TGCCTGACAAGTTGAGACAT |
|  | TLR07_1R2 | GCGGGATCAGTGGTCTAAGA |
| TLR09A | TLR09_1F | CCAAAAGGAGCATCACAGGT |
|  | TLR09_1R | ACGGAATGCATCAAGGTGGA |
|  | TLR09_2F | CTTCACCAGGGACCTCTTCA |
|  | TLR09_2R | CGGGAAAGTCTCAGTGGTCT |
|  | TLR09_3F | TGATGAAAGTGGCATTGAACC |
|  | TLR09_3R | CAGAGATTCAAGCACAGTTGG |
|  | TLR09_4F | AGGGTTTGGGTCACAGCTTT |
|  | TLR09_4R | GCCTTCAAGATATGTGCCCC |
|  | TLR09_5F | TAGAGTGCACATGTGAGGCA |
|  | TLR09_5R | TGCATCCACACTAGCCATCA |
|  | TLR09_1R2 | CCTTCATGGCATGGGAAAGA |
|  | TLR09_2F3 | TCCACCTTRATGCATTCCGT |
|  | TLR09_2R3 | AAAGCTGTGACCCAAACCCT |
|  | TLR09_3F2 | AGCTAATTCTTTCCCATGCCA |
|  | TLR09_3R2 | TATCCTTCCTTCCCTGCCAC |
| TLR09B | TLR09B_1F | ACCCTCCCTCAATTTCTGCC |
|  | TLR09B_1R | AGGTTGGTTAAAGGCACAAACA |
|  | TLR09B_2F | CCAACGATGTGACCAYGCTT |
|  | TLR09B_2R | TGRCTCCCGTTTAATCGTTGG |
|  | TLR09B_3F | CAGAAACCTCRCCACTAAGC |
|  | TLR09B_3R | TGCAAGCAGAGAAGAATTCCT |
| TLR13 | TLR13_1F | GCAGACGCCTCATCCAAAA |
|  | TLR13_1R | GTGGGTTCTGATCAAAGACCA |
|  | TLR13_2F | TGACCAAATTATCCACACTGCA |
|  | TLR13_2R | CTCACATACGTAAAGGTGTCCC |
|  | TLR13_3F | TCCCTTGAGACCCTTTACCT |
|  | TLR13_3R | CCAAATTGGCAAAAGCGTCC |

Table S1 continued

| TLR gene | primer name | sequence 5'->3' |
| --- | --- | --- |
| TLR13 | TLR13_4F | GCTGTTTAAAGGGCTGCAGA |
|  | TLR13_4R | TGTAGAATTCCCCAGGCAGA |
|  | TLR13_5F | ACTCTGTTGACCATGATTGCC |
|  | TLR13_1R2 | TGTCTTCTCCGAAAGCAGGT |
|  | TLR13_2F2 | TGCCAAATATCCAGCACYTG |
|  | TLR13_2R2 | ATAGGGAGAGGTAGCGGAGA |
|  | TLR13_4R_4769 | TGTMGAATTCCCCAGGCAGA |
|  | TLR13_5R_4769 | GCTGATGTAGGCGTGGTTTC |
|  | TLR13_4F2 | TCCAAAACCTTTCACTGCTCC |
|  | TLR13_4R2 | CCCCAGGMAGAAAATCYCT |
| TLR21 | TLR21_1F | ATGGCTTCAGGAACTGCATC |
|  | TLR21_1R | AAAGTGAGGTGCTGCTCAGG |
|  | TLR21_2F | GGCTGGACCTGTCTGAGAAC |
|  | TLR21_2R | ATGGGGGCTTTGATCTGATT |
|  | TLR21_3F | GCTGGACAACAACCTGCTAA |
|  | TLR21_3R | AAGGGTCAACGTCACAGCTT |
|  | TLR21_4F | AGACGCTTGACCTGGAGAAC |
|  | TLR21_4R | GGAGGCCACGTGATGTAAGT |
|  | TLR21_5F | TATGTCTTCCGAGCCTGGTT |
|  | TLR21_5R | TGGAAGCTCTCTGAGGGTGT |
|  | TLR21_1R2 | TGYAGCCCCATTCCWGAATA |
|  | TLR21_2F2 | TGKCTTCCCTYAACACCTCRA |
|  | TLR21_2R2 | AGGCCTTTGAAGAWRTTGGGT |
| TLR22A | TLR22_1F | TAACAGCCCAGAGGGTAGCA |
|  | TLR22_1R | TCCACTAGATTGTCGGAGTCTG |
|  | TLR22_2F | TTGGATACCTCATACGTTGGTG |
|  | TLR22_2R | CAATGGACTGCCAATGTTGT |
|  | TLR22_3F | TCAGGCACTTAGCAGATTGG |
|  | TLR22_3R | AATGCAGCACTTGGGTTTTC |
|  | TLR22_4F | TACGTGCAGTGGGAAATCAA |
|  | TLR22_4R | CCACGCAAAATTGGGTTATT |
|  | TLR22_1R2 | GGTCCAACATTTGTAAAGATGGC |
|  | TLR22_2F2 | TCTGCTCTTTCCAACCAAATMT |
|  | TLR22_2R2 | GGTGTAAGTAAAAGTGGTTGGGT |
| TLR22B | TLR22B_1F | GTAGTCCTGGTCCTGGTTCC |
|  | TLR22B_1R | CCAGGGAACAGCAAGAAAGG |
|  | TLR22B_2F | GGCATTAACGTGACCAGCAA |
|  | TLR22B_2R | CGCCAAGTTAAGGGTTTKCA |
|  | TLR22B_3F | TCTTCCCAGTTTCAGGCCYT |
|  | TLR22B_3R | TTGKTTATCACAGCTGCACG |

Table S2. Model fit and model choice. The best M2GFA model in bold.

| model | p-value | marginal density | Bayes factor | posterior probability |
| --- | --- | --- | --- | --- |
| M1 | 0.008 | 3.09E-25 | 4.58E-08 | 4.58E-08 |
| M1GF | 0.336 | 1.16E-24 | 1.72E-07 | 1.72E-07 |
| M1GFA | 0.757 | 2.25E-25 | 3.78E-08 | 3.78E-08 |
| M2W | 0.014 | 1.75E-22 | 2.60E-05 | 2.60E-05 |
| M2N | 0.18 | 4.78E-19 | 0.0762 | 0.071 |
| M2GFW | 0.518 | 1.29E-24 | 1.92E-07 | 1.92E-07 |
| M2GFN | 0.832 | 3.28E-20 | 0.00489 | 0.005 |
| M2GFA | 0.313 | 6.24E-18 | 12.211 | 0.924 |

Table S3. Prior and posterior distributions for M2GFA model parameters. Ne – effective population size; Lm – *Lissotriton montandoni;*NePre – effective population size before the demographic change; Lvg – *L. vulgaris graecus*; MLm – number of migrants per generation into *L. montandoni*, MLvg – number of migrants per generation into *L. v. graecus*; u – mutation rate per generation, per bp; r – recombination rate per generation, between adjacent sites; TLm – time in generations, with assumed generation time of 4 years since the demographic change in *L. montandoni*; TLvg – time since the demographic change in *L. v. graecus*; Tsplit – time of divergence between *L. montandoni* and *L. v. graecus*; lo95q – lower 95 percentile of the posterior distribution; hi95q – upper 95 percentile of the posterior distribution.

|  | prior | | posterior | | | |
| --- | --- | --- | --- | --- | --- | --- |
|  | min | max | mode | mean | lo95q | hi95q |
| Ancestral Ne | 1.0E+04 | 3.16E+06 | 7.9E+05 | 2.0E+05 | 1.3E+04 | 2.6E+06 |
| Lm Ne | 1.0E+04 | 1.0E+07 | 5.8E+05 | 4.8E+05 | 5.9E+04 | 3.41E+06 |
| Lm NePre | 1.0E+04 | 3.16E+06 | 3.6E+04 | 9.2E+04 | 1.2E+04 | 1.9E+06 |
| Lvg Ne | 1.0E+04 | 1.0E+07 | 6.0E+05 | 5.1E+05 | 5.9E+04 | 3.65E+06 |
| Lvg NePre | 1.0E+04 | 3.16E+06 | 4.1E+04 | 1.0E+05 | 1.1E+04 | 1.93E+06 |
| MLm | 1.0E-2.5 | 5 | 0.76 | 2.04 | 0.11 | 4.67 |
| MLvg | 1.0E-2.5 | 5 | 0.81 | 2.00 | 0.11 | 4.65 |
| u | 5.0E-10 | 5.0E-08 | 9.95E-09 | 1.76E-08 | 1.49E-09 | 4.4E-08 |
| r | 1.0E-10 | 1.0E-08 | 3.98E-09 | 4.95E-09 | 4.48E-10 | 9.55E-09 |
| TLm | 1.0E+03 | 1.0E+06 | 2.2E+05 | 3.8E+05 | 2.6E+04 | 8.9E+05 |
| TLvg | 1.0E+03 | 1.0E+06 | 2.4E+05 | 4.1E+05 | 2.6E+04 | 9.0E+05 |
| Tsplit | 1.0E+05 | 2.5E+06 | 1.75E+06 | 1.46E+06 | 3.2E+05 | 2.42E+06 |

Table S4. List of codons under positive selection identified by the gammamap method.

| Gene | Codon | Pr(γ>0)^a^ | AA change | Domain | Gene | Codon | Pr(γ>0) | AA change | Domain |
| --- | --- | --- | --- | --- | --- | --- | --- | --- | --- |
| *L. montandoni* | | | | | *L. v. graecus* | | | | |
| TLR02 | 284 | 0.97 | I-L |  | TLR02 | 302 | 0.79 | G-E |  |
| TLR02 | 287^#^ | 0.98 | C-S |  | TLR02 | 369 | 0.81 | L-V | LRR |
| TLR02 | 290 | 0.86 | Y-N |  | TLR02 | 385 | 0.81 | E-K |  |
| TLR05L | 348^*^ | 0.84 | F-L | LRR^b^ | TLR02 | 430^#^ | 0.80 | M-I |  |
| TLR05L | 486^*^ | 0.88 | S-Y |  | TLR03 | 296 | 0.85 | T-S | LRR |
| TLR09A | 76 | 0.76 | Y-D/G |  | TLR03 | 352^*^ | 0.91 | H-R | LRR |
| TLR09B | 105^*^ | 0.75 | L-F |  | TLR03 | 395 | 0.83 | T-K |  |
| TLR09B | 317^*^ | 0.87 | D-A | LRR | TLR03 | 499 | 0.84 | Y-H | LRR |
| TLR21 | 287 | 0.76 | S-P |  | TLR03 | 529 | 0.85 | E-K |  |
| TLR21 | 292 | 0.79 | Y-H |  | TLR07 | 92 | 0.83 | I-L |  |
| TLR21 | 294 | 0.79 | S-G | LRR | TLR07 | 285 | 0.78 | S-R |  |
| TLR21 | 351 | 0.77 | A-V |  | TLR09A | 41 | 0.76 | C-G/S |  |
| TLR21 | 397^#^ | 0.81 | T-I | LRR | TLR09B | 98^*^ | 0.93 | A-D |  |
| TLR21 | 398 | 0.81 | H-Q | LRR | TLR09B | 104^*^ | 0.93 | K-E |  |
| TLR22B | 439 | 0.81 | H-R |  | TLR09B | 284^*^ | 0.92 | R-I | LRR |
|  |  |  |  |  | TLR09B | 343^*^ | 0.92 | M-V |  |
|  |  |  |  |  | TLR09B | 376^*^ | 0.93 | K-E |  |
|  |  |  |  |  | TLR09B | 379 | 0.89 | E-K |  |
|  |  |  |  |  | TLR09B | 409^*#^ | 0.92 | P-S |  |
|  |  |  |  |  | TLR09B | 468^*^ | 0.92 | T-A |  |
|  |  |  |  |  | TLR13 | 59 | 0.82 | D-A | LRR |
|  |  |  |  |  | TLR13 | 218 | 0.82 | A-D | LRR |
|  |  |  |  |  | TLR13 | 232 | 0.83 | M-L |  |
|  |  |  |  |  | TLR21 | 134^*^ | 0.92 | E-Q | LRR |
|  |  |  |  |  | TLR21 | 161^*^ | 0.92 | A-T | LRR |
|  |  |  |  |  | TLR21 | 273^*^ | 0.93 | R-H | LRR |
|  |  |  |  |  | TLR21 | 294 | 0.81 | S-G | LRR |
|  |  |  |  |  | TLR21 | 295^*^ | 0.96 | S-N | LRR |
|  |  |  |  |  | TLR21 | 435^*^ | 0.92 | E-K | LRR |
|  |  |  |  |  | TLR21 | 455^*^ | 0.92 | A-S | LRR |
|  |  |  |  |  | TLR22A | 65 | 0.87 | R-K | LRR |
|  |  |  |  |  | TLR22B | 36 | 0.75 | T-A |  |
|  |  |  |  |  | TLR22B | 247 | 0.85 | L-I |  |

* codons fixed within population.

^#^ deleterious effect of codon replacement as identified by PROVEAN, the remaining amino acid substitutions were identified as neutral.

^a^ estimated posterior probability of positive selection.

^b^LRR – leucine-rich repeats.

 Figure S1. Location of primers used for amplification of *Lissotriton* TLR genes. Thick blue line – coding sequence, thin blue lines – untranslated regions.

 Figure S2. Geographic location of samples used to assess TLR polymorphism species-wide (grey circles) and in two population samples: *L. monatandoni* (red triangles) and *L. vulgaris graecus* (blue triangles).

Figure S3. A Bayesian tree depicting relationship between members of the TLR05 family. Both genes encoding the full form, and the form lacking transmembrane and TIR domains are shown.

Figure S4. Expression of *Lissotriton* TLR genes in liver and spleen. FPKM - Fragments Per Kilobase Per Million Fragments Mapped.

Figure S5. Nucleotide diversity in the species-wide dataset*;* π - overall, π_S_ – synonymous and π_N_ – nonsynonymous nucleotide diversity. Dashed line shows average for markers located in 3’UTR transcript regions of 62 genes.
